# Multi-operator Self-exclusion as a Harm Reduction Measure in Problem Gambling: Retrospective Clinical Study on Gambling Relapse Despite Self-exclusion

**DOI:** 10.2196/37837

**Published:** 2022-08-19

**Authors:** Anders Håkansson, Gunny Åkesson

**Affiliations:** 1 Clinical Research Unit Competence Center Addiction, Malmö Addiction Center Region Skåne Malmö Sweden; 2 Division of Psychiatry Department of Clinical Sciences Lund Lund University Lund Sweden

**Keywords:** web-based gambling, gambling disorder, behavioral addiction, self-exclusion, addiction, gambling, prevalence, harm reduction, abstain, problem gambling

## Abstract

**Background:**

Voluntary self-exclusion from gambling is a common harm reduction option for individuals with gambling problems. Multi-operator, nationwide self-exclusion services are rare, and a system introduced in the highly web-based gambling market of Sweden is a rare and recent example. However, where web-based casino gambling and web-based betting are the predominate gambling types in those seeking treatment, the risk of breaching one’s own self-exclusion through overseas web-based operators may also be high.

**Objective:**

This study aims to assess the prevalence of a nationwide *Spelpaus* (“gambling break”) self-exclusion and the prevalence of gambling despite self-exclusion in patients seeking treatment for gambling disorder in 2021.

**Methods:**

Health care documentation of recent treatment seekers (January 1 through September 1, 2021, N=85) in a Swedish treatment facility was reviewed for data regarding problematic gambling types reported, history of self-exclusion, and history of breaching of that self-exclusion.

**Results:**

Common problem gambling types were web-based casino gambling (49/74, 66%) and sports betting (19/74, 26%). The majority who participated in this study (62/85, 73%) were men. All women reported web-based casino gambling. Self-exclusion through *Spelpaus* was common (60/74, 81%). Among self-excluders, gambling despite self-exclusion was common (41/60, 68%), most commonly on unlicensed gambling websites.

**Conclusions:**

The nationwide, multi-operator self-exclusion service of Sweden appears to reach many patients with a gambling disorder. However, the remaining gambling options in an web-based gambling setting present a major challenge despite self-exclusion. The recent data calls for further treatment efforts and potential improvements in services aiming to help voluntary self-excluders abstain from gambling.

## Introduction

Gambling disorder is an addictive condition known to have major deteriorating effects on individuals’ mental health and social and financial situation [1]. One of the interventions available in the management of problem gambling is the individual’s own possibility to self-exclude from gambling, that is, to limit one’s own access to a web-based gambling site or a physical gambling venue, to prevent one’s own relapse into gambling. This is a method available in many land-based or web-based gambling settings [2,3] and may serve as a viable option for gamblers who perceive severe problems and who may or may not seek formal treatment and for whom treatment seeking may often be too limited [4].

In Sweden, a previously unreported type of self-exclusion service, *Spelpaus*, meaning “gambling break” in direct translation, was introduced in the gambling legislation from January 2019, allowing gamblers to self-exclude from all licensed gambling operators operating either via the internet or physically in Sweden, including a large number of operators in web-based and land-based casinos, sports and horse racing betting, web-based card games, web-based lotteries, and web-based bingos. The self-exclusion period can be chosen to be either 1 month, 3 months, 6 months, or a year, and although each period is discontinued automatically, one can enter the system at any time to prolong it. *Spelpaus* is accessed at a specific website [5] belonging to the Swedish Gambling Authority, which is a national government authority, and the provision of this service is therefore independent of the individual gambling operators. Hitherto, it has been reported that a majority of problem gamblers likely have not self-excluded, although a substantial number of them do, and a substantial number of individuals without gambling problems also may choose to self-exclude in this system [6]. *Spelpaus* is promoted by gambling operators through radio, television, newspapers, and advertisements on the internet, as well as by preventive and therapeutic institutions. As the service is frequently mentioned in advertisement breaks in popular media, it may have relatively good potential to be heard of by both gamblers and nongamblers in the population.

Any system based on voluntary self-exclusion from gambling is associated with a certain risk of breaching the exclusion, as part of an addictive gambling pattern with lowered self-control. However, the risk of breaching the self-exclusion is very little described for web-based gambling services. Also, such data are virtually unavailable for the present type of nationwide self-exclusion services that involve a large number of gambling types and operators. In a population survey in Sweden, among online gamblers responding to a web-based survey, 38% of those who had ever self-excluded via this Swedish national self-exclusion service had gambled at some point during the exclusion period [7]. Likewise, recent data have shown that in a policy-based measure excluding gamblers from each gambling operator after reaching a maximum loss limit at that operator, it was common for gamblers with moderate risk or problem gamblers to gamble on other operators; this indicates a risk that individuals with intense gambling patterns may migrate to other operators in case of responsible gambling measures putting a limit to their gambling [8]. Thus, given this type of possible migration between operators, it can be suspected that even patients with gambling disorder who choose voluntary self-exclusion may be at risk of switching to other operators thereafter.

Sweden has a highly web-based gambling market, with web-based casino gambling and sports betting representing the vast majority of patients seeking treatment [9] and the vast majority of commercial advertisements seen on television [10]. One major task of policy making in the area has been to exclude unlicensed overseas gambling operators from the market to keep Swedish gambling within the legal operators, which are likely to follow government regulations including self-exclusion [11]. After the introduction of the *Spelpaus* self-exclusion service, a policy debate has addressed the risk of breaching this self-exclusion [7], typically because of the risk of migrating to overseas gambling services, and thereby, limiting the efficiency of self-exclusion. Based on this debate, it is important for policy making in the field to assess whether it is common for patients with gambling disorder to self-exclude from gambling, and whether it is common for them to gamble despite this self-exclusion. For this reason, this subanalysis was carried out as a substudy from a larger retrospective chart review study in a gambling disorder treatment unit. This substudy aimed to evaluate the prevalence of *Spelpaus* self-exclusion and the prevalence of *Spelpaus* breaching in treatment-seeking patients with gambling disorder.

## Methods

### Study Participants

This study is a substudy of a larger systematic, retrospective hospital chart study. The treatment episodes included are from the regional gambling disorder unit of Region Skåne, where both authors are employed, within the public health care services. This facility has a responsibility for the assessment and treatment of gambling disorder in the whole county of Skåne in southern Sweden, a county with around 1.4 million inhabitants. Other treatment providers in the county include the social services of local municipalities, which may offer different types of interventions for treatment-seeking clients with problem gambling, as well as the voluntary, nongovernmental peer support provided by local patient organizations. The Region Skåne health care unit is specialized in gambling and assesses treatment-seeking adults aged 18 years or older with gambling problems, typically on a diagnostic level corresponding to the World Health Organization diagnostic code of gambling disorder (F63.0 [12]). This treatment facility has been previously described in publications discussing the gambling types and comorbidity in patients with gambling disorder [9] and lack of evident changes in treatment seeking at the facility after the onset of the COVID-19 pandemic [13]. Predominate gambling types at the facility are web-based casino gambling, which is also the gambling type most commonly seen in televised gambling advertising in Sweden [10], and sports betting. The majority of patients (80%) are men, and 58% are diagnosed with another psychiatric comorbidity in parallel with the gambling disorder [9]. The facility opened in December 2015 and has therefore been operating both before and after the formal introduction of gambling into the treatment responsibilities of Swedish health care and social service institutions in January 2018.

### Study Procedures

This is a substudy originating from a larger one that aimed to study characteristics and changes in patients seeking treatment at the facility before and after the introduction of gambling into the Swedish addiction treatment legislation, before and after the outbreak of COVID-19, and before and after the introduction of specific gambling legislations by the Swedish government due to COVID-19. The COVID-19–related aspect of this study was the suspicion during the COVID-19 pandemic that societal changes during the pandemic may be associated with increased gambling patterns, at least in individuals with gambling problems [14]. Given the specific picture of the data collected from the most recent year (2021) and given the political and media attention to the role of the *Spelpaus* self-exclusion service in current trends in web-based gambling, a specific subanalysis was carried out, involving only the treatment episodes from January 1 through September 1, 2021.

This subanalysis includes all patients who applied for treatment and received an appointment at the unit from January 1 through September 1, 2021, that is, during a period when both authors were employed by and actively working at the facility as the medically responsible physician (AH) and as one of the therapists (GÅ). The second author of the paper (GÅ) reviewed each record of health care documentation from the facility. Data extracted for this descriptive analysis include gender, type of problematic gambling reported, whether the patient had reported national *Spelpaus* self-exclusion upon treatment seeking, and whether the patient reported gambling (and the type of modality) despite this self-exclusion.

### Ethics Approval

The overall study was approved by the Swedish Ethics Review Authority (file number 2021-03636) on August 11, 2021. Also, the specific subanalysis included in this paper along with its specific aim was approved in an amendment to the same authority (file number 2022-00911-02) on February 22, 2022. In line with the ethics approval, no information or consent from patients was required in this retrospective chart review study.

### Statistical Methods

Results are reported as descriptive frequencies, including absolute numbers and percentages, and gambling despite using *Spelpaus* was compared between women and men, using the Fisher exact test with significance defined as *P*<.05.

## Results

A total of 85 individuals were included in the study. Among them, 62 (73%) were men and 23 (27%) were women. A total of 7 patients (2 men and 5 women) did not start the formal treatment. Full data were not found in the chart review in 4 cases (4 men). Further data were available for 74 individuals, that is, 56 (76%) men and 18 (24%) women.

Problematic gambling types reported were web-based casino gambling (49/74, 66%), sports betting (19/74, 26%), and other land-based gambling (1/74, 1%); the gambling type was missing in 5 cases (7%). Web-based casino gambling was reported by 100% of women (18/18) and 55% of men (31/56), whereas sports betting was reported by 34% (19/56) of men and none of the women.

In total, 81% (60/74) had self-excluded through *Spelpaus*, 4% (3/74) had not, and data were unavailable from the chart text in 15% (11/74) of the cases. All 3 individuals who did not report self-exclusion were men. Out of those having self-excluded, 68% (41/60) reported having gambled despite using *Spelpaus*, that is, 63% of men (27/43) and 82% of women (14/17), without any significant gender difference (*P*=.22). Among clients reporting gambling despite using *Spelpaus*, this was reported to occur on unlicensed gambling websites in 68% of the cases (28/41), and gambling with somebody else’s identity or unlicensed gambling in land-based venues in 22% of the cases (9/41); the information was missing for 10% (4/41) of the cases.

## Discussion

This substudy aimed to provide an up-to-date observation regarding the dissemination and feasibility of a harm reduction service (*Spelpaus*) in use since 2019 in the Swedish gambling market. This unique, nationwide, multi-operator self-exclusion service, operating in a highly web-based gambling market, has theoretically faced with a substantial challenge from the hypothetical competition with overseas web-based operators that are not included in this self-exclusion service. This analysis demonstrates that among patients seeking help for gambling disorder in recent months, adherence to the self-exclusion service is very common; but among those who self-excluded, as many as 68% had gambled despite self-exclusion. Thus, given the fact that these patients are actively seeking treatment for a severe gambling problem, it must be assumed that their gambling problem was associated with the gambling activity that breached the self-exclusion.

Therefore, this subanalysis, from a larger clinical study, can be seen as an alert for improved measures against gambling in people with gambling problems who seek measures to control their gambling patterns. It can be concluded that in the present setting, where web-based gambling is highly predominant among patients with gambling disorder [9], this self-exclusion service is insufficient for these patients to cope with their addictive behavior. Structured treatment of the condition is therefore required over and above this self-exclusion service, but also, policy makers may need to consider technical or legal possibilities to expand the self-exclusion possibility beyond the present setting to gambling markets that are web-based and cannot be expected to respect geographical borders.

A nationwide, multi-operator self-exclusion system appears to attract people with severe gambling problems, as a clear majority of treatment seekers in this study reported having enrolled in this system. This can be seen as a major merit of this self-exclusion system, such that it reaches the individuals for whom it is intended. It goes beyond this study, which was carried out in a clinical setting, to analyze whether the service also successfully reaches individuals with subdiagnostic but significant gambling problems, as they also must be considered as a target group of this harm reduction service.

Our study clearly indicates a major challenge of this type of self-exclusion service in a setting where web-based gambling is common, as individuals with gambling disorders may be attracted to overseas gambling operators, which are unlicensed in Sweden, and therefore, beyond the jurisdiction of its gambling regulations. Policy makers should address further technical and judiciary possibilities of promoting self-exclusion from other gambling operators and not only those operating within the country’s geographical setting. Additionally, treatment providers and preventive institutions should address the fact that self-exclusion from gambling is an insufficient measure for individuals with gambling disorders, who also may need structured treatment for their addictive behavior.

In addition to the findings regarding the self-exclusion service, it must be noted that a clear majority of the patients seeking treatment during these 8 months had a problematic gambling pattern associated with web-based casinos. In particular, this was the only problematic gambling type reported by the women in the study, pointing to the potential risk of more female gamblers being recruited to problem gambling due to this modern gambling type. It confirms the previously demonstrated addiction potential of web-based casino gambling, as this is the most common gambling type in patients seeking treatment in this study and previous clinical studies [9]; however, this type of gambling is in fact used by a very small minority of gamblers in the general population [15].

This study, a timely subanalysis from a larger project, aimed to inform stakeholders in the area about an ongoing situation in a gambling market where several different measures of responsible gambling and preventive strategies have been tested. The study results also suggest the need for further, more in-depth studies about how gambling self-exclusion is perceived by users and whether the choice to self-exclude, in gamblers who breach their self-exclusion, may still be beneficial or have negative effects. Such further research may partly require qualitative studies to describe in more detail the considerations made by gamblers in relation to self-exclusion. Also, the use of objective gambling data or financial loan data in those who self-exclude, in relation to their exclusion date and in relation to their breaching, may highlight more closely the potential effects of the self-exclusion system. However, in contrast to its timely findings, this study clearly has some limitations. The study was conducted in a single treatment facility and only for a period of 8 months, which means that the study sample is limited in size. This clearly limits the potential for statistical calculations and the generalizability of findings to other settings. In addition, the number of study variables derived from a retrospective analysis of medical records, intended not primarily for research but for routine treatment purposes, is limited.

In conclusion, this brief report confirms the fact that patients with gambling disorder are successfully reached by the relatively unique Swedish nationwide self-exclusion service, but it also clearly demonstrates the current challenge of self-exclusion in a modern, highly digitalized gambling market. Therefore, although the study supports the self-exclusion service used here, it also concludes that formal treatment, further preventive work, and more advanced services for self-exclusion from unlicensed gambling operators are important. In addition, the overwhelming majority of web-based casino gamblers in a single treatment facility also confirms the previously reported addiction potential of this gambling type.
